# Surgical treatment option for incidental azygos vein aneurysm: a case report

**DOI:** 10.1093/jscr/rjaf089

**Published:** 2025-03-04

**Authors:** Angel Guan, Aldin Malkoc, Kevin Perez, Kendall Vignaroli, So Un Kim, Danielle Cremat, Ashley Wang, Lawrence Kong

**Affiliations:** The Division of General Surgery, Department of Surgery, Arrowhead Regional Medical Center, 400 N Pepper Ave, Colton, CA 92324, United States; The Division of General Surgery, Department of Surgery, Arrowhead Regional Medical Center, 400 N Pepper Ave, Colton, CA 92324, United States; The Division of General Surgery, Department of Surgery, Arrowhead Regional Medical Center, 400 N Pepper Ave, Colton, CA 92324, United States; The Division of General Surgery, Department of Surgery, Arrowhead Regional Medical Center, 400 N Pepper Ave, Colton, CA 92324, United States; The Division of General Surgery, Department of Surgery, Arrowhead Regional Medical Center, 400 N Pepper Ave, Colton, CA 92324, United States; The Division of General Surgery, Department of Surgery, Arrowhead Regional Medical Center, 400 N Pepper Ave, Colton, CA 92324, United States; The Division of General Surgery, Department of Surgery, Arrowhead Regional Medical Center, 400 N Pepper Ave, Colton, CA 92324, United States; The Division of Thoracic Surgery, Department of Surgery, Kaiser Permanente Fontana Medical Center, 9961 Sierra Ave, Fontana, CA 92335, United States

**Keywords:** azygos vein aneurysm, mediastinal mass

## Abstract

Azygos vein aneurysms (AVA) are rare pathologies of the thorax that can mimic a posterior mediastinal mass. Patients with AVA may be asymptomatic, or present with chest pain, pulmonary embolisms, and acute rupture. Currently, there are no standardized guidelines for treatment. Management varies from interval surveillance, prophylactic oral anticoagulation or anti-platelet, endovascular embolization, and/or surgical resection. We present the case of a 45-year-old female who was found to have an incidental azygos vein aneurysm. She was asymptomatic and underwent robotic assisted mediastinal mass resection with no intra-operative or post-operative complications.

## Introduction

Azygos vein aneurysms (AVA) are uncommon and infrequently diagnosed. There are currently no guidelines on optimal treatment or standard of care because the literature of this topic is scarce. Currently, there are ˂100 case reports on this diagnosis and treatment options vary with each one [1]. Based on the current literature, the etiology of AVA are categorized into idiopathic, pressure or volume overload, or traumatic. Clinical presentation can vary from being asymptomatic to acute rupture. Other symptoms include thromboembolism, mediastinal mass effect, and pulmonary artery hypertension [2]. The diagnosis can be made with computed or magnetic resonance tomography (CT) scans and can often be diagnosed incidentally when getting these scans for other reasons [3]. Treatment for AVA vary from active surveillance to anticoagulation or antiplatelet, to surgical resection [4]. Since there are no guidelines, the treatment is often based on the patient’s co-morbidities, age, and preference. To avoid future complications of embolisms and fatal rupture, we recommend surgical resection if the patient is an appropriate surgical candidate with life expectancy of at least 5 years [5]. We present a case of a 45-year-old female with an incidentally found AVA who underwent robotic thoracoscopic resection of the AVA with full recovery and no complications.

## Case presentation

**Figure 1:**
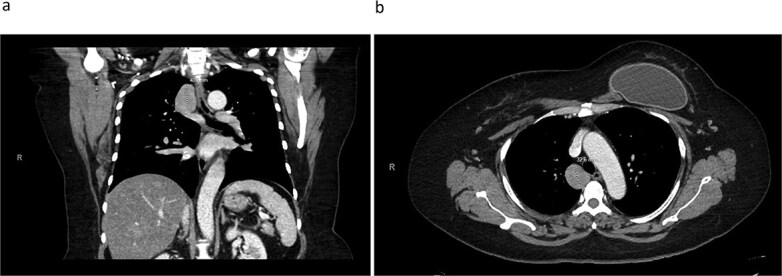
CT thorax revealing a 4.1 × 3.3 cm azygos vein aneurysm. a) Coronal view. b) Transverse view.

**Figure 2:**
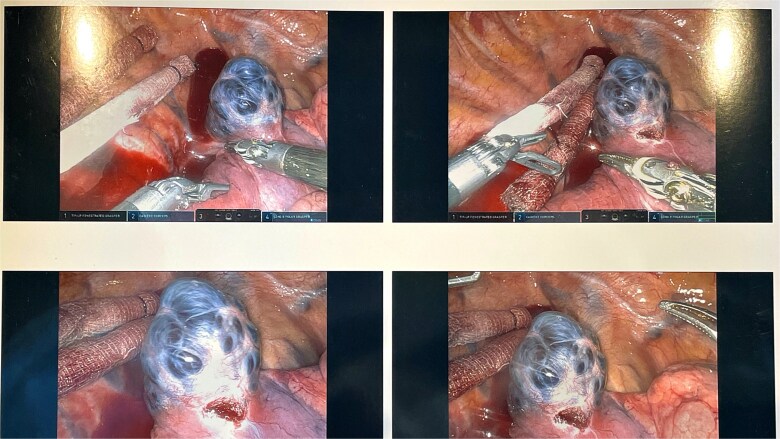
The intra-operative findings included a hemorrhagic multi-cystic appearing lesion which was connected to the azygos vein as well as the apex of the right lung.

**Figure 3:**
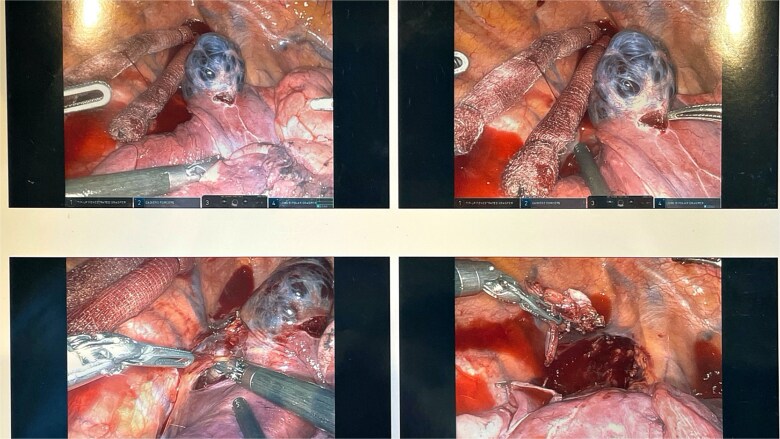
This lesion was isolated with electrocautery and blunt dissection; proximal and distal control of the azygos vein were transected with a surgical stapler.

A 45-year-old female with past medical history of hypertension and unspecified connective tissue disorder presented to the emergency room with leg pain, chest pain, and dyspnea with initial blood pressure of 197/138 due to medication noncompliance. Due to the constellation of symptoms combined with severe hypertension, CT chest was ordered to rule out aortic dissection. The scan did not show aortic dissection but incidentally found 4 cm azygos vein aneurysm. The patient had resolution of symptoms, blood pressure was control with medications, and she has been followed outpatient for the azygos vein aneurysm with conservative management of daily aspirin and annual CT thorax scans for 4 years. Four years later, she presented to our clinic with no symptoms of chest pain, dyspnea, cough, and so on but wanted to discuss surgical options. Her most recent CT thorax revealed a stable azygos vein aneurysm measuring 4 cm (Fig. 1). After discussing the risks and benefits of surgery, the patient consented to surgical resection. The patient then underwent robotic assisted mediastinal mass resection with no complications. The intra-operative findings included a hemorrhagic multi-cystic appearing lesion which was connected to the azygos vein as well as the apex of the right lung (Fig. 2). This lesion was isolated with electrocautery and blunt dissection; proximal and distal control of the azygos vein were transected with a surgical stapler and chest tube was placed (Fig. 3). Postoperatively, patient recovered well, chest tube was removed, and she was discharged home on postoperative day one. Surgical pathology was benign and revealed a portion of lung parenchyma with associated dilated blood vessels. She is doing well and has no cardiac or pulmonary symptoms 2 years after surgery.

## Discussion

The exact cases of AVA are unknown and difficult to identify with many patients. Case reports have described an idiopathic incidentally discovered congenital anomaly to be the cause [6]. We believe this may be the cause for our patient as well. In addition, our patient also had an unspecified connective tissue disorder, diagnosed after her surgery, which most likely also contributed to the formation of her AVA. Other causes of AVA may include increased central venous pressure, portal hypertension, inferior vena cava occlusion, heart failure, or trauma [6–8]. AVA is a rare pathology that can be managed with conservative treatment for many years without complications. However, thoracoscopic resection is a preferred treatment to prevent complications such as thrombosis and rupture. We present a case where both conservative for 4 years and subsequent surgical management was successful for our patient. She was diagnosed with a connective tissue disorder which makes her high risk for complications such as thrombosis and rupture. She made a full recovery from the surgery and continues to do well 2 years post operatively.

## References

[1] Kreibich M, Siepe M, Grohmann J, et al. Aneurysms of the azygos vein. J Vasc Surg Venous Lymphat Disord 2017;5:576–86. 10.1016/j.jvsv.2016.12.012. 28624000

[2] Mehta M, Towers M. Computed tomography appearance of idiopathic aneurysm of the azygos vein. Canadian Assoc Radiol J = Journal l'Association canadienne des radiologistes 1996;47:288–90. 8696998

[3] Choo JY, Lee KY, Oh SJ, et al. Azygos vein aneurysm mimicking paratracheal mass: dynamic magnetic resonance imaging findings. Balkan Med J 2013;30:111–5. 10.5152/balkanmedj.2012.095. 25207080 PMC4116017

[4] Ling X, Yu R, Fang L, et al. Thoracoscopic approach to the resection of idiopathic azygos vein aneurysm: a case report. J Cardiothorac Surg 2022;17:163. 10.1186/s13019-022-01908-5. 35725603 PMC9210694

[5] Kurihara C, Kiyoshima M, Asato Y, et al. Resection of an azygos vein aneurysm that formed a thrombus during a 6-year follow-up period. Ann Thorac Surg 2012;94:1008–10. 10.1016/j.athoracsur.2012.01.086. 22579898

[6] Tujo CA, Jesinger RA. Azygous vein aneurysm (AVA): a case report. J Clin Diagn Res 2017;11:TD03–5. 10.7860/JCDR/2017/20945.9421. PMC5376782 28384961

[7] He J, Mao H, Li H, et al. A case of idiopathic azygos vein aneurysm and review of the literature. J Thorac Imaging 2012;27:W91–3. 10.1097/RTI.0b013e318217272d. 21659895

[8] Savu C, Melinte A, Balescu I, et al. Azygos vein aneurysm mimicking a mediastinal mass. In Vivo 2020;34:2135–40. 10.21873/invivo.12019. 32606194 PMC7439865

